# Detection and characterization of human bocaparvovirus in children with and without acute gastroenteritis in African-descendant community of Northern Brazil

**DOI:** 10.1371/journal.pone.0333474

**Published:** 2025-11-04

**Authors:** Endrya Socorro Fôro Ramos, Patrícia Santos Lobo, Delana Andreza Melo Bezerra, Jane Haruko Kaiano, Consuelo Silva de Oliveira, Eliete da Cunha Araújo, Danielle Rodrigues de Deus, Joana D’Arc Pereira Mascarenhas, Sylvia de Fátima dos Santos Guerra, Luana da Silva Soares

**Affiliations:** Section of Virology, Evandro Chagas Institute, Ananindeua, Pará, Brazil; Università degli Studi di Parma: Universita degli Studi di Parma, ITALY

## Abstract

Human bocaparvovirus (HBoV) is an emerging virus with worldwide distribution, may be associated with cases of acute gastroenteritis (AGE). To date, four types of HBoV have been characterized: HBoV1, HBoV2, HBoV3 and HBoV4. This study aimed to investigate HBoV in asymptomatic and symptomatic children for AGE from a Quilombola community located in Northern, Brazil, during April 2008 to September 2010. A total of 300 fecal specimens were collected and viral genomic DNA was extracted, amplified using the PCR assay, and subject to sequencing to determine HBoV genotype. HBoV was detected in 11.3% (34/300) of the samples, 12.50% (12/96) from symptomatic and 10.78% (22/204) asymptomatic children. Co-detection with other enteric viruses was reported in 20.6% (7/34) of specimens. Three genotypes of HBoV were detected with the most predominance of HBoV1 (64.7%), followed by HBoV4 (20.6%) and HBoV2 (14.7%). Phylogenetic analysis demonstrated that Brazilian HBoV are closely related with strains from South America, Asia, Africa and Oceania. This is the first description of HBoV in a Quilombola community in Brazil, and this study highlights the ability of the virus to remain in silent circulation in the community, reinforce the need for active monitoring in order to avoid problems public health futures.

## Introduction

Acute gastroenteritis (AGE) is defined as an acute infection in the gastrointestinal tract whose etiology involves different pathogens such as viruses, bacteria, protozoa, helminths and fungi. Viruses being the most prevalent etiological agents, corresponding to 50% of AGE cases [1,2]. Among the viral agents, the most prominent are norovirus (NoV), rotavirus A (RVA), and enteric human adenovirus (HAdV) [3,4]. However, in many cases, around 40% of the etiology of the infection is unknown [1,5].

Human bocavirus (HBoV) was first identified in Stockholm, Sweden in 2005 [6] initially in respiratory tract infections, and then detected in patients with AGE [7–9]. Meanwhile, its contribution to the etiology of AGE cases, as well as the circulating types associated with this condition have not yet been elucidated [1,3,10].

HBoV belongs to *Parvoviridae* family, *Parvovirinae* subfamily and *Bocaparvovirus* genus [11]. It is a non-enveloped virus with icosahedral symmetry and a linear genome consisting of single-stranded deoxyribonucleic acid (ssDNA) of approximately 5.3 kb. Its genomes present three Open Reading Frames (ORF) that encode four proteins: NS1, a non-structural viral protein involved in the replication process and with endonuclease function; NP1, a non-structural nuclear phosphoprotein related to the apoptosis process; VP1 and VP2, structural proteins responsible for viral capsid formation. These proteins are used for viral detection, with NS1 and NP1 regions constituting the most conserved region and VP1 and VP2 showing higher variability [1,7,12].

Regarding classification, HBoV are classified into two species, that have the potential to infect different hosts, such as mammals and non-human primates. The species *Primate bocaparvovirus 1*; includes the HBoV genotypes called HBoV-1 and HBoV-3, the species *Primate bocaparvovirus 2*; includes HBoV-2 and HBoV-4 [6–9]. In addition to these species, the species *Primate bocaparvovirus 3* has also recently been proposed; which includes non-human bocaparvoviruses primate [13]. Most studies associate HBoV-1 with the respiratory tract while the HBoV-2, HBoV-3 and HBoV-4 species are more related to the gastrointestinal tract. However, there is a need for further studies to elucidate diseases caused by different types of HBoV [9,14].

The present study aimed to describe HBoV species circulating in children, with and without acute gastroenteritis in an African-descendant semi-closed community (Abacatal Quilombola Community) located in Pará State, Northern Brazil.

## Materials and methods

### Ethics

The Ethics Committee on Human Research of the Evandro Chagas Institute (IEC-CEPH) granted ethical approval to our study under number 06199719.9.0000.0019 in April 2019. The ethical consent form was applied to the subjects of this research. Initially, the study team held meetings with community members, such as community leaders, health visitors and school directors, in order to obtain a better understanding of the study area and to inform them about the research objectives. Written informed consent was signed by parents or guardians of the children during the fecal specimen collection. The study was carried out in accordance with the Declaration of Helsinki of 1975 (https://www.wma.net/what-we-do/medical-ethics/declaration-of-helsinki/), revised in 2013.

### Study population and sample collection

Fecal samples were collected from children aged between 0 and 12 years (average 5 years) during April 2008 to September 2010 through regular weekly visits in the community of Abacatal, located in the municipality of Ananindeua, belonging to the metropolitan region of Belém, capital of the state of Pará, Amazon region, Brazil. The map this community located in Ananindeua had demonstrated by Aragão et al [15]. This retrospective study analyzed epidemiological data from May 2019 to May 2020 using a total of 300 fecal samples. Samples from children under 12 years of age were included and samples that had exhausted fecal specimens were excluded from the analysis.. Of these, 63% (189/300) were from children up to 5 years of age, 50.7% (152/300) were male and 68% (204/300) were from the non-diarrheal group. These samples were tested for other gastroenteric viruses like Rotavirus (RVA), Norovirus (NoV) Human Adenovirus and Human Astrovirus (HAstV) in previous studies [15], and the results obtained were used to assess the presence of monoinfection or co-infection with HBoV.

### Detection and molecular characterization of HBoV

Viral DNA was extracted from a 10% fecal suspension (Tris HCl/Ca^++^ 0,01M pH 7,2) using a guanidine isothiocyanate/silica method carried out according to the previously described protocol [16]. The nucleic acid was stored at – 20°C. Molecular detection was performed using polymerase chain reaction (PCR) followed by nested-PCR, with specific primers, aiming to amplify the gene that encodes the VP1/VP2 protein The nucleic acid was stored at −20°C. Molecular detection was performed by polymerase chain reaction (PCR) followed by nested PCR, with specific primers, aiming to amplify the gene encoding the VP1/VP2 protein. The PCR primers AK-VP-F1 (5’-CGCCGTGGCTCCTGCTCT −3’) and AK-VP-R1 (5’-TGTTCGCCATCACAAAAGATGTG-3’) were used in the PCR while the primers AK-VP-F2 (5’GGCTCCTGCTCTAGGAAATAAAGAG-3’) and AK-VP-R2 (5’-CCTGCTGTTAGGTCGTTGTTGTATGT-3’) were used in the nested PCR, using the conditions previously described [8].

The amplicons obtained in Nested-PCR were purified using a commercial kit (PureLink PCR Purification kit, Invitrogen^TM^, CA, USA), according to the manufacturer recommendations. Subsequently, purified DNA was subjected to a sequencing reaction, in both directions, using a Big Dye Terminator Cycle Sequencing Ready Reaction Kit^®^ (v.3.1) (Applied Biosystems, CA, USA) and an ABI Prism Model 3130xl DNA Sequencer (Applied Biosystems, Foster City, USA).

The partial sequences of the VP1/VP2 genes were assembled and edited using the Geneious program (v. 9), and then aligned using the Mafft algorithm (v.7) with HBoV 1, 2, 3 and 4 sequences deposited in the database [17,18]. For phylogenetic analysis, the maximum likelihood method was adopted using the FastTree v software. 2.1.11, including the robust GTR+Gamma+F substitution model and with bootstrap reliability testing configuring 1,000 consensus replicates in order to obtain reproducible results and provide greater reliability to the clades [19]. The nucleotide sequences identified in this study were deposited in GenBank (https://www.ncbi.nlm.nih.gov/) with accession numbers OR338847-OR338880.

### Statistical analysis

All data were analyzed using descriptive statistics, processed using the PAST.Uio software through bivariate analysis of variables (age, sex and year of sample collection) in the two research groups, using the chi-square test (χ^2^) with p value ≤ 0.05 considered statistically significant [20].

## Results

### HBoV detection

Overall, HBoV was identified in 11.3% (34/300) of samples with HBoV-positivity rates of 12.5% (12/96) and 10.8% (22/204) recorded in diarrheic and non-diarrheic children, respectively. Regarding sex, similar HBoV occurrence was observed in females (55.9%, 19/34) and males (44.1%, 15/34). Concerning age group, the most affected were children aged under five years old represented 52.9% (18/34) while those aged 6–12 years old represented 47.1% (16/34). During the years 2008–2010, a significant increase in the identification of HBoV was reported. No statistical significance was observed in the analyzed groups. This data is shown in Table 1 below.

**Table 1:** The association between epidemiological aspects and HBoV infection in children from an African-descendant semi-closed community located in Pará State, Northern Brazil.

Epidemiological Variables	Diarrheic		Non-diarrheic		Total		*p value*
	n	%	n	%	n	%	
**Sex**							
Female	5	41.7	14	63.6	19	55.9	0.21
Male	7	58.3	8	36.4	15	44.1	
**Age group**							
≤ 5 years	8	66.7	10	45.5	18	52.9	0.23
≥ 6–12 years	4	33.3	12	54.5	16	47.1	
**Period**							
2008	2	16.7	3	13.6	5	14.7	0.07
2009	1	8.3	10	45.5	11	32.4	
2010	9	75.0	9	40.9	18	52.9	

### Co-infection with HBoV and enteric viruses

Co-infection involving HBoV and HAdV were detected in 14.7% (5/34) of the samples, while 5.9% (2/34) presented HBoV and RVA. In all cases, children were symptomatic for AGE (S1 Table).

### Genotyping and phylogenetic analysis of HBoV

**Fig 1:**
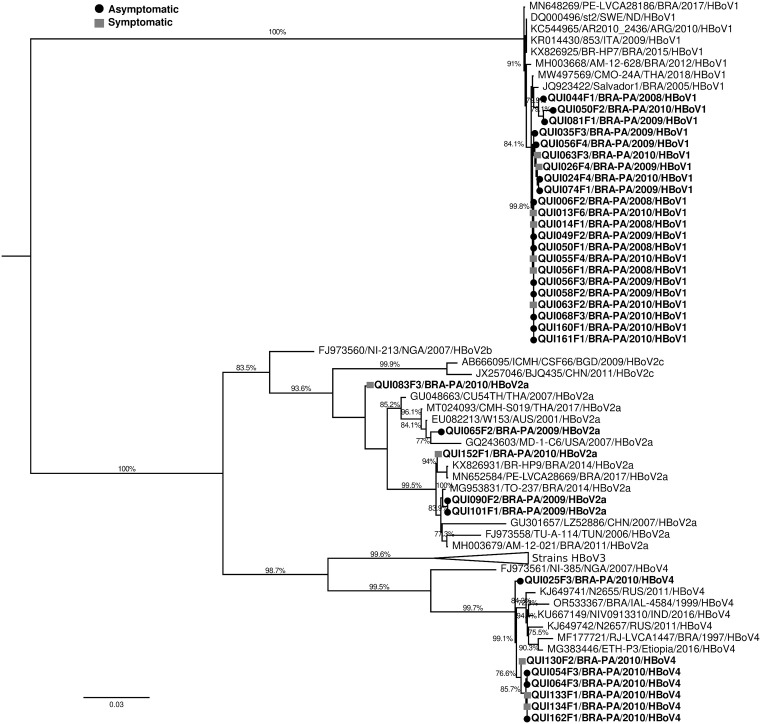
Phylogenetic relationship of HBoV “Quilombola” samples with other RVA sequences from Brazil and the world, based on the nucleotide sequence of the VP1/VP2 partial genes. Samples of the present study were highlighted in bold and identified according to “Sample name/country of origin/year of isolation/genotype”. The circular symbol represents the group of asymptomatic samples and the square symbol represents the group of symptomatic samples. The values shown (>70%) indicate support for 1000 *bootstrap* values.

Genotyping of all specimens revealed the circulation of HBoV-1 (64.7%, 22/34), HBoV-2 (14.7%, 5/34) and HBoV-4 (20.6%, 7/34). Based on the phylogenetic inference of the VP1/VP2 partial gene, 22 strains of HBoV-1 genotype were grouped with isolates from Brazil, Thailand, Argentina and European strains that circulated from 2005 to 2018 with bootstrap support values ranging from 78.3% to 100% and with a high nucleotide similarity (99–100%). Five strains were classified as HBoV-2, lineage 2a, clustering with strains from several countries around the world such as Brazil, United States, Australia, Thailand, China, and Tunisia, circulated between 2001 and 2017, with bootstrap support value ranging from 79.1% to 99.6%, and nucleotide similarity ranging 93.6 to 99.8% among them. Concerning HBoV-4 clade, seven “Quilombola” strains clustered with specimens from Brazil, Russia, India, and Ethiopia detected between 1997 and 2016 with nucleotide similarity scores ranging from 98.2% to 99.4%. These results were showed in dendrogram of Fig 1 below.

## Discussion

HBoV has been described in distinct locations around the world in symptomatic and asymptomatic patients for AGE [9,21–25]. These findings suggest HBoV as an emerging pathogen worldwide, and the investigation of this viral agent is extremely important in order to understand its molecular and epidemiological aspect, given that it can be an important viral agent of gastrointestinal infection [24,25].

In the present study, the occurrence of HBoV was evidenced circulating in children with and without symptoms of AGE from an African descendant community in Brazil in an overall positivity of 11.3%. Others studies conducted in Amazon region reported similar frequencies. Trindade et al. [26] detected HBoV in 10% of children up to 5 years with or without AGE symptoms in Acre. Leitão et al. [27] detected HBoV in 14.2% of younger Amazonian children with AGE. It is worth highlighting that HBoV frequency worldwide in children with AGE is changeable, since low occurrence were related in Korea (0.8%) [28], EUA (1.4%) [21] and China (1.4%) [29] and higher prevalence have already been described in Brazil (41.9%) [30].

With respect HBoV occurrence in symptomatic and asymptomatic AGE groups, in the present analysis the frequencies were similar. Even though, some studies demonstrate a higher detection rate in symptomatic children for AGE [9,31]. This fact could be related to the increase in viral load usually observed in patients with symptoms, which contributes to the dissemination and detection of this pathogen in fecal samples.

Regarding age groups, the presence of HBoV was found in both age groups analyzed in Table 1, with a slight increase in children under 5 years old (52.9%). Similar studies conducted in different countries attribute a higher frequency of HBoV in this age group, in which the majority of AGE cases are also observed and reinforcing that HBoV could plays an important pathogen in childhood diarrhea and indicated that young children are prone to HBoV infection [32–36].

Although some authors associate a higher incidence of HBoV in male sex [24,35,37], no significant differences of HBoV detection among sex were observed in the present study. This highlights the importance of conducting further studies to deepen the understanding of the epidemiological aspects of this viral agent.

HBoV co-infection with other enteric viruses has been reported worldwide. In this study, HBoV was co-detected with other enteric viruses that are involved in AGE in a relatively low frequency compared to data from several studies that correlated HBoV with other enteric viruses [1,38,39]. These findings suggest that this agent may play a collaborative role in the development of gastrointestinal tract infections. However, further research is needed since this pathology can be caused by distinct pathogenic agents.

Phylogenetic analysis revealed a significant predominance of HBoV-1. This specie was also observed by different authors in Brazil [30,36,40]. Although this genotype is more commonly associated with respiratory tract infections, it is also identified in fecal samples due its excretion after infection for months [10,41]. However, there is no evidence confirming whether its presence in feces stems from a gastrointestinal tract infection or arises as a consequence of a respiratory tract infection. Therefore, further research is needed to elucidate the association of HBoV-1 with AGE [23,24].

Besides the predominance of HBoV-1 in this study, other species were found, such as HBoV-2 and HBoV-3, commonly identified in stools samples from children with AGE symptoms [41]. All sequences classified as HBoV-2 clustered within the clade of HBoV-2a, with no sequences attributed to HBoV-2b or HBoV-2c. Furthermore, this study identified a significant rate of HBoV-4 strains (20.6%), once this specie is rarely detected. In Brazil, HBoV‐4 was previously identified in fecal samples only in two studies. Sousa et al. [25] detected HBoV-4 in a fecal sample from a hospitalized child with a soft tissue tumor in the submandibular region in 2015 and Viana et al. [36] reported this specie in one fecal sample collected in 1999 understand its natural history in patients with diarrhea.

To date, this is the first report of HBoV circulation in an African descendant community in Brazil. Therefore, it was difficult to compare the present data with other investigations about HBoV in this population. Therefore, it is worth highlighting that HBoV has circulated in this semi-closed community, which may explain the high frequency of HBoV-4 in this population and not provide the propagation of this specie outside the community.

A limitation of the study was the absence of epidemiological characteristics to establish the features of HBoV infection in this population. Another restriction was the lack of screening of other enteric pathogens (e.g., bacteria and parasites) to elucidate the etiologic role of HBoV in AGE end the impact of these other enteric agents on the etiology of AGE.

## Conclusion

This study highlights epidemiological and molecular features of HBoV infection in a Quilombola community. This emphasizes the importance of investigation to understand the distribution of this virus, contributing to knowledge of molecular epidemiology and relevance of circulating types, aiming to prevent potential public health issues.

## Supporting information

S1 Table Description of epidemiological variables and HBoV genotypes detected in the symptomatic or asymptomatic group of infection in children from an African-descendant semi-closed community located in Pará State, Northern Brazil. (DOC)
