# Supplementary material for: Detection and characterization of human bocaparvovirus in children with and without acute gastroenteritis in African-descendant community of Northern Brazil
Source: PLoS One. 2025 Nov 4;20(11):e0333474. doi: 10.1371/journal.pone.0333474 (PMC12585048; doi:10.1371/journal.pone.0333474)
Supplement: S1 Table — (DOC) [file pone.0333474.s001.doc]

**S1 Table.** **Description of epidemiological variables and HBoV genotypes detected in the symptomatic or asymptomatic group of infection in children from an African-descendant semi-closed community located in Pará State, Northern Brazil.**

| **Strain** | **Age** | **Gender** | **Year** | **Genotype** | **Group** | **Co-infections (viruses)** | **Accession numbers** | **URL** |
| --- | --- | --- | --- | --- | --- | --- | --- | --- |
| QUI-006F2 | 5 | M | 2008 | HBOV-1 | Asymptomatic | - | OR338847 | <https://www.ncbi.nlm.nih.gov/nuccore/OR338847> |
| QUI-013F6 | 2 | M | 2010 | HBOV-1 | Symptomatic | Adenovirus enteric | OR338848 | <https://www.ncbi.nlm.nih.gov/nuccore/OR338848> |
| QUI-014 F1 | 1 | F | 2008 | HBOV-1 | Symptomatic | - | OR338849 | <https://www.ncbi.nlm.nih.gov/nuccore/OR338849> |
| QUI-024 F4 | 8 | F | 2010 | HBOV-1 | Asymptomatic | - | OR338850 | <https://www.ncbi.nlm.nih.gov/nuccore/OR338850> |
| QUI-025 F3 | 7 | F | 2010 | HBOV-4 | Asymptomatic | - | OR338851 | <https://www.ncbi.nlm.nih.gov/nuccore/OR338851> |
| QUI-026 F4 | 4 | F | 2009 | HBOV-1 | Symptomatic | Adenovirus enteric | OR338852 | <https://www.ncbi.nlm.nih.gov/nuccore/OR338852> |
| QUI-035 F3 | 12 | F | 2009 | HBOV-1 | Asymptomatic | - | OR338853 | <https://www.ncbi.nlm.nih.gov/nuccore/OR338853> |
| QUI-044 F1 | 3 | M | 2008 | HBOV-1 | Asymptomatic | - | OR338854 | <https://www.ncbi.nlm.nih.gov/nuccore/OR338854> |
| QUI-049 F2 | 6 | M | 2009 | HBOV-1 | Asymptomatic | - | OR338855 | <https://www.ncbi.nlm.nih.gov/nuccore/OR338855> |
| QUI-050 F1 | 6 | F | 2008 | HBOV-1 | Asymptomatic | - | OR338856 | <https://www.ncbi.nlm.nih.gov/nuccore/OR338856> |
| QUI-050 F2 | 1 | F | 2010 | HBOV-1 | Asymptomatic | - | OR338857 | <https://www.ncbi.nlm.nih.gov/nuccore/OR338857> |
| QUI-054 F3 | 4 | F | 2010 | HBOV-4 | Asymptomatic | - | OR338858 | <https://www.ncbi.nlm.nih.gov/nuccore/OR338858> |
| QUI-055 F4 | 4 | M | 2010 | HBOV-1 | Symptomatic | Adenovirus enteric | OR338859 | <https://www.ncbi.nlm.nih.gov/nuccore/OR338859> |
| QUI-056 F1 | 4 | F | 2008 | HBOV-1 | Symptomatic | - | OR338860 | <https://www.ncbi.nlm.nih.gov/nuccore/OR338860> |
| QUI-056 F3 | 9 | F | 2009 | HBOV-1 | Asymptomatic | - | OR338861 | <https://www.ncbi.nlm.nih.gov/nuccore/OR338861> |
| QUI-056 F4 | 6 | F | 2009 | HBOV-1 | Asymptomatic | - | OR338862 | <https://www.ncbi.nlm.nih.gov/nuccore/OR338862> |
| QUI-058 F2 | 7 | M | 2009 | HBOV-1 | Asymptomatic | - | OR338863 | <https://www.ncbi.nlm.nih.gov/nuccore/OR338863> |
| QUI-063 F2 | 4 | M | 2010 | HBOV-1 | Symptomatic | Adenovirus enteric | OR338864 | <https://www.ncbi.nlm.nih.gov/nuccore/OR338864> |
| QUI-063 F3 | 8 | M | 2010 | HBOV-1 | Symptomatic | - | OR338865 | <https://www.ncbi.nlm.nih.gov/nuccore/OR338865> |
| QUI-064 F3 | 6 | F | 2010 | HBOV-4 | Asymptomatic | - | OR338866 | <https://www.ncbi.nlm.nih.gov/nuccore/OR338866> |
| QUI-065 F2 | 10 | M | 2009 | HBOV-2 | Asymptomatic | - | OR338867 | <https://www.ncbi.nlm.nih.gov/nuccore/OR338867> |
| QUI-068 F3 | 8 | M | 2010 | HBOV-1 | Asymptomatic | - | OR338868 | <https://www.ncbi.nlm.nih.gov/nuccore/OR338868> |
| QUI-074 F1 | 5 | F | 2009 | HBOV-1 | Asymptomatic | - | OR338869 | <https://www.ncbi.nlm.nih.gov/nuccore/OR338869> |
| QUI-081 F1 | 2 | F | 2009 | HBOV-1 | Asymptomatic | - | OR338870 | <https://www.ncbi.nlm.nih.gov/nuccore/OR338870> |
| QUI-083 F3 | 1 | F | 2010 | HBOV-2 | Symptomatic | Adenovirus enteric | OR338871 | <https://www.ncbi.nlm.nih.gov/nuccore/OR338871> |
| QUI-090 F2 | 8 | M | 2009 | HBOV-2 | Asymptomatic | - | OR338872 | <https://www.ncbi.nlm.nih.gov/nuccore/OR338872> |
| QUI-101 F1 | 7 | F | 2009 | HBOV-2 | Asymptomatic | - | OR338873 | <https://www.ncbi.nlm.nih.gov/nuccore/OR338873> |
| QUI-130 F2 | 4 | M | 2010 | HBOV-4 | Symptomatic | Rotavirus | OR338874 | <https://www.ncbi.nlm.nih.gov/nuccore/OR338874> |
| QUI-133 F1 | 12 | M | 2010 | HBOV-4 | Symptomatic | - | OR338875 | <https://www.ncbi.nlm.nih.gov/nuccore/OR338875> |
| QUI-134 F1 | 3 | M | 2010 | HBOV-4 | Symptomatic | - | OR338876 | <https://www.ncbi.nlm.nih.gov/nuccore/OR338876> |
| QUI-152 F1 | 6 | F | 2010 | HBOV-2 | Symptomatic | Rotavirus | OR338877 | <https://www.ncbi.nlm.nih.gov/nuccore/OR338877> |
| QUI-160 F1 | 6 | M | 2010 | HBOV-1 | Asymptomatic | - | OR338878 | <https://www.ncbi.nlm.nih.gov/nuccore/OR338878> |
| QUI-161 F1 | 1 | F | 2010 | HBOV-1 | Asymptomatic | - | OR338879 | <https://www.ncbi.nlm.nih.gov/nuccore/OR338879> |
| QUI-162 F1 | 4 | F | 2010 | HBOV-4 | Asymptomatic | - | OR338880 | <https://www.ncbi.nlm.nih.gov/nuccore/OR338880> |
